
JOURNAL INFORMATION
==============================
NLM Title Abbreviation: Wirtschaftsdienst
Journal ID: Wirtschaftsdienst
NLM Journal ID: 101086612

Wirtschaftsdienst (Hamburg, Germany : 1949)

ISSN: 0043-6275

ARTICLE INFORMATION
==============================
PMCID: PMC8857628
PMID: 35221391
DOI: 10.1007/s10273-022-3118-3
Article ID: 3118
Article version: 1
Subjects: Ökonomische Trends

Demografische Alterung führt zu einem stark sinkenden Erwerbspersonenpotenzial

Demographic Ageing Leads to a Sharply Declining Labour Force Potential

Fuchs Johann Johann.Fuchs@iab.de

Söhnlein Doris Doris.Soehnlein@iab.de

Weber Birgit Brigitte.Weber@iab.de

grid.425330.3 0000 0001 1931 2061 Prognosen und gesamtwirtschaftliche Analysen (MAKRO), Institut für Arbeitsmarkt- und Berufsforschung (IAB), Regensburger Str. 104, 90478 Nürnberg, Deutschland
Electronic publication date: 2022 Feb 19
Print publication date: 2022
Volume: 102
Issue: 2
First page: 148
Last page: 150
Copyright: © Der/die Autor 2022
License: Dieser Artikel wird unter der Creative Commons Namensnennung 4.0 International Lizenz veröffentlicht (creativecommons.org/licenses/by/4.0/deed.de).
Open Access wird durch die ZBW — Leibniz-Informationszentrum Wirtschaft gefördert.
License URL: https://creativecommons.org/licenses/by/4.0/

Keywords: J11, J21, J22
issue-copyright-statement: © The Author(s) 2022

==============================

Aktuell wird trotz der Coronapandemie intensiv über einen demografisch bedingten künftigen Fachkräftemangel diskutiert, wobei schon jetzt viele offene Stellen mangels Bewerber:innen nicht besetzt werden können (Kubis, 2021). Die Folgen wären fatal: Betriebe könnten schließen oder ins Ausland abwandern und es drohen Finanzierungsprobleme in der Sozialversicherung. Die Fachkräftediskussion geht dabei von einem Rückgang des Arbeitskräfteangebots aus, also der Zahl an Arbeitskräften, die den Betrieben potenziell zur Verfügung steht. Aufgrund des seit langer Zeit bekannten negativen demografischen Trends ist mit einem stark sinkenden Arbeitskräftepotenzial zu rechnen. Die aktuellste Projektion des Instituts für Arbeitsmarkt- und Berufsforschung (Fuchs et al., 2021) zeigt, mit welchen Einbrüchen beim Arbeitskräfteangebot künftig zu rechnen ist.

Über viele Jahre hat ein steigendes Erwerbspersonenpotenzial den Aufschwung am Arbeitsmarkt gestützt. Unmittelbar vor der Covid-19-Pandemie zählte die deutsche Statistik 45,1 Mio. Erwerbstätige, 1,4 Mio. Erwerbslose und 0,9 Mio. Personen in der „Stillen Reserve“, die manchmal als verdeckte Arbeitslosigkeit betrachtet wird. Zusammen ergab das 2019 ein Erwerbspersonenpotenzial von fast 47,4 Mio. Personen – rund 2,9 Mio. mehr als zur Jahrtausendwende. Hohe Zuzüge aus dem Ausland und eine zunehmende Erwerbsbeteiligung von Frauen und Älteren machten dies trotz eines negativen demografischen Trends möglich. Doch schon vor der Pandemie kam der Zuwachs beim Erwerbspersonenpotenzial ins Stocken (Bauer et al., 2021). Rein rechnerisch ergibt sich das Erwerbspersonenpotenzial aus der Bevölkerung[1] und deren Erwerbsbeteiligung[2], wobei Letztere vom Alter, dem Geschlecht und der Staatsangehörigkeit abhängt. Abbildung 1 zeigt die Entwicklung des Erwerbspersonenpotenzials bei unterschiedlichen Annahmen zur künftigen Erwerbsbeteiligung und zum Wanderungsgeschehen. Damit lässt sich diese Entwicklung in die Einflussfaktoren Demografie, Verhalten (Erwerbsquoten) und Migration zerlegen. Die unterste Linie in der Abbildung 1 stellt ein Szenario mit dem rein demografischen Effekt dar. Dieser resultiert aus dem Bevölkerungsrückgang und vor allem aus der Alterung. Szenario 1 blendet also das Wanderungsgeschehen völlig aus der Betrachtung aus (und entspricht damit einer hypothetischen Situation, in der niemand nach Deutschland zieht, aber auch niemand das Land verlässt). Außerdem basiert es auf der heutigen Erwerbsbeteiligung, d.h. konstanten Erwerbsquoten. Aufgrund der aktuell gegebenen Altersstruktur würde das Erwerbspersonenpotenzial in den kommenden Jahren stark schrumpfen. Bis 2035 beträgt der demografische Effekt -7,2 Mio. Personen (-15 %). Der Rückgang setzt sich nach 2035 fort, sodass das Erwerbspersonenpotenzial 2060 mit 31,3 Mio. Arbeitskräften um gut 16,1 Mio. unter dem Stand im Ausgangsjahr 2020 läge (-34 %).

Realistischerweise ist von einer weiter steigenden Erwerbsbeteiligung der Frauen und Älteren auszugehen. Die Erwerbsquoten deutscher Frauen liegen in den mittleren Jahrgängen aktuell bereits bei 90 %. Ausländerinnen haben dagegen um bis zu 20 Prozentpunkte niedrigere Erwerbsquoten. Höhere Erwerbsquoten wären bei ihnen sicher denkbar und dieser substanzielle Unterschied nimmt in der Projektion auch etwas ab. Es bleibt trotzdem eine erhebliche Differenz zur Erwerbsbeteiligung deutscher Frauen. Bei den Älteren ist als Folge der Rentengesetzgebung (Rente mit 67) mit einem Anstieg der Erwerbsquoten zu rechnen. Zudem dürfte bei einem wachsenden Anteil an Akademiker:innen – mit einem in der Regel längeren Verbleib im Erwerbsleben – die Erwerbsquote weiter steigen. In der vorliegenden Projektion (Szenario 2) steigt die Erwerbsquote bei den 60- bis 64-jährigen Männern von derzeit 74 % bis 2030 auf 82 %; bei den 60- bis 64-jährigen Frauen von nicht ganz 67 % auf 70 %. Weiter zählen in der Projektion 2020 fast 30 % der 65- bis 69-jährigen Männer zum Erwerbspersonenpotenzial; 2030 werden es gut 52 % sein. Bei den Frauen dieser Altersgruppe steigt die Erwerbsquote von derzeit 20 % auf 34 %. Nach 2030 verharren die Erwerbsquoten der Älteren weitgehend auf dem dann erreichten Niveau. In einem zweiten Szenario – ebenfalls ohne Wanderung – werden diese zu erwartenden Steigerungen der Erwerbsquoten sowohl bei Frauen als auch bei Älteren angenommen. Aufgrund der höheren Erwerbsquoten

1 Basisjahr der Bevölkerungsprojektion ist 2020. Die Ausgangsbevölkerung wurde deterministisch mit der Komponenten-Kohorten-Methode nach Alter und Geschlecht fortgeschrieben. Um dem besonderen Einfluss der Zuwanderung und der unterschiedlichen Erwerbsbeteiligung gerecht zu werden, differenziert das Modell zusätzlich nach Nationalität – nach Deutschen und Nichtdeutschen (Fuchs und Söhnlein, 2005).

2 Die Erwerbsbeteiligung wird nach Altersgruppen, Geschlecht und Deutschen/Nichtdeutschen vorausgeschätzt. Alle Berechnungen basieren auf den Erwerbsquoten des Mikrozensus plus der hinzugeschätzten Stillen Reserve, wofür eine aktuelle Schätzung vorliegt (Fuchs und Weber, 2021). Erwerbsquoten plus Stille-Reserve-Quoten ergeben Potenzialerwerbsquoten, die zur sprachlichen Vereinfachung im Text als Erwerbsquoten bezeichnet werden.

Abbildung 1

**Entwicklung des Erwerbspersonenpotenzials**

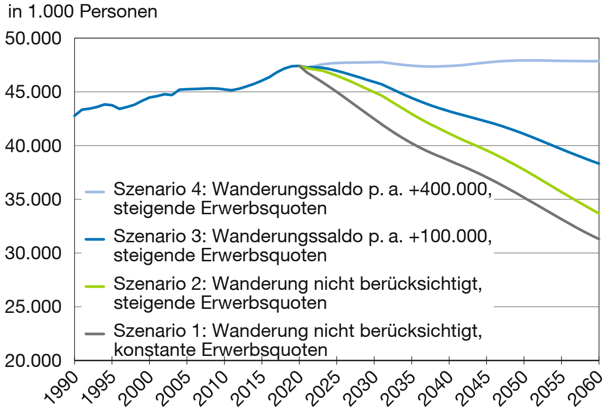

Quelle: IAB-Kurzbericht 25/2021 (Fuchs et al., 2021).

verläuft der Abwärtstrend bei Szenario 2 weniger extrem als bei Szenario 1. Im Unterschied zu einer rein demografischen Betrachtung ergibt sich ein positiver verhaltensbedingter Effekt, der sich bis 2060 auf ca. 2,4 Mio. an zusätzlichen Arbeitskräften beläuft. Trotzdem sinkt das Erwerbspersonenpotenzial bis 2035 um knapp 4,5 Mio. Da sich die Zuwächse aufgrund steigender Erwerbsquoten bis 2060 abschwächen, ergibt sich ein Rückgang des Erwerbspersonenpotenzials bis 2060 von 13,7 Mio. (-29 %) gegenüber 2020.

Der Migrationseffekt auf das Erwerbspersonenpotenzial hängt entscheidend vom Wanderungssaldo ab. Dargestellt werden zwei Wanderungsvarianten. Beide gehen von den gleichen Erwerbsquoten wie Szenario 2 aus. Bei einer jährlichen Nettozuwanderung von 100.000 Personen sinkt das Erwerbspersonenpotenzial von 47,4 Mio. 2020 auf 44,4 Mio. 2035 und auf 38,3 Mio. 2060 (Szenario 3). Bis 2060 würde das Erwerbspersonenpotenzial bei dieser (angesichts der aktuellen Zahlen eher niedrigen) Nettozuwanderung um 9,1 Mio. Personen gegenüber 2020 schrumpfen (-19 %). Für eine vollständige Kompensation bräuchte es eine jährliche Nettozuwanderung von 400.000 Personen (Szenario 4). Daraus würde bis 2060 ein Wanderungseffekt von 14,2 Mio. Erwerbspersonen resultieren. Mit dem erwähnten Verhaltenseffekt von 2,4 Mio. würde dies den demografischen Effekt langfristig leicht übertreffen: Wir errechnen für 2060 ein Erwerbspersonenpotenzial von fast 47,9 Mio. Personen.

Die im Vergleich zu den letzten Jahren vor der Pandemie niedrige Nettozuwanderung von 100.000 Personen pro Jahr von Szenario 3 bewerten wir als eine durchaus realistische, langfristige Möglichkeit für künftige Wanderungsströme. Ein wesentlicher Grund dafür ist unter anderem das sinkende Wanderungspotenzial aus den Hauptherkunftsländern der EU-Zuwanderung durch den dort ebenfalls wirkenden demografischen Wandel. Außerdem steigt bei höheren Zuzügen die Zahl der in Deutschland lebenden ausländischen Bevölkerung, und damit steigen auch deren Fortzüge. Nicht zu vergessen, dass auch viele Personen mit deutscher Nationalität das Land verlassen. Deren negativer Wanderungssaldo senkt die Gesamtzuwanderung. Aus der Komponentenzerlegung – Demografie, Verhalten und Migration – wird die überragende Bedeutung der Demografie für die künftige Entwicklung des Arbeitskräfteangebots deutlich: Durch eine höhere Erwerbsbeteiligung und bei eher niedrigen Wanderungsannahmen lässt sich der Effekt der demografischen Alterung nicht kompensieren.

Der demografische Trend wirkt sich nicht nur auf die absolute Größenordnung des Erwerbspersonenpotenzials aus, sondern führt zwischenzeitlich zu dessen starker Alterung. Die Migration der tendenziell eher jüngeren Menschen beeinflusst zwar das Ausmaß der demografischen Alterung, hält sie aber wohl nicht auf. Nach den vorliegenden Berechnungen dürften am Arbeitsmarkt künftig weniger Erwerbspersonen jüngeren und mittleren Alters zur Verfügung stehen. In Abbildung 2 wird dieser Trend für ausgewählte Jahre dargestellt. 2020 waren 9,4 Mio. Erwerbspersonen jünger als 30 Jahre. Im Szenario 3 mit einem Wanderungssaldo von 100.000 sind es 2035 gut 8,5 Mio. und 2060 nur noch 7,2 Mio. Die Zahl der 30- bis 49-jährigen Erwerbspersonen sinkt bei diesem Szenario von 19,6 Mio. 2020 auf 18,8 Mio. 2035 und anschließend bis 2060 auf 16,3 Mio. Die Zahl der älteren Arbeitskräfte ist in der Vergangenheit stark gestiegen: Das Potenzial der 50-jährigen und älteren Erwerbspersonen lag 2000 bei 10,3 Mio. Im Basisjahr der Projektion zählten wir bereits 18,4 Mio. ältere Erwerbspersonen. Bei einer Nettozuwanderung von 100.000 Personen und steigenden Erwerbsquoten dürfte sich ihre Zahl bis 2035 auf 17,1 Mio. verringern. 2060 umfasst diese Altersgruppe beim Szenario 3 nur noch 14,8 Mio. Erwerbspersonen. Der Grund für dieses Absinken ist die Alterung der Baby-Boomer-Generation, von der immer mehr in Rente gehen. In Abbildung 2 ist zu erkennen, dass die Gruppe der 65-Jährigen und Älteren noch längere Zeit größer wird, ganz langfristig gesehen aber auch abnimmt. Bei Szenario 4 mit einem Wanderungssaldo von 400.000 Personen würde sich der Umfang der jüngeren und mittleren Altersjahrgänge des Erwerbspersonenpotenzials gegenüber 2020 kaum ändern oder sogar geringfügig steigen, der Rückgang bei den Älteren wäre allerdings deutlich geringer: 2035 wären es 17,7 Mio. und 2060 knapp 18,0 Mio. 50-jährige und ältere Erwerbspersonen. Auch die Zugewanderten unterliegen der Alterung.

Abbildung 2
**Altersstruktur des Erwerbspersonenpotenzials**

Annahmen: Wanderungssaldo p. a. +100.000, steigende Erwerbsquoten
in Mio. Personen

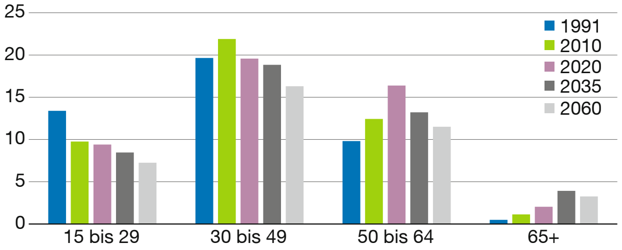

Quelle: eigene Darstellung (Fuchs, Söhnlein und Weber, 2021).

Denkbar, obgleich nicht sehr realistisch, wären noch höhere Erwerbsquoten. Dazu wurden drei Simulationen mit sehr optimistischen Annahmen hinsichtlich der Erwerbsquoten von deutschen Frauen (Vereinbarkeit), Ausländerinnen (Integration) und Älteren (Renten) gerechnet. Die Simulation zur Vereinbarkeit bildet eine vollständige Angleichung der Erwerbsquoten deutscher Frauen an die deutscher Männer von 30 bis 59 Jahren ab. Die Simulation zur Integration zeigt den Effekt einer besseren Arbeitsmarktintegration der in Deutschland lebenden Ausländerinnen. Dabei werden für die 15- bis 59-jährigen Ausländerinnen die gleichen Erwerbsquoten wie bei deutschen Frauen angenommen. Der Effekt eines längeren Verbleibs Älterer im Erwerbsleben geht nach Fuchs und Weber (2020) von einer Verschiebung der Erwerbsquoten aus: Für die Gruppe der 60- bis 64-Jährigen wird künftig die Erwerbsquote der 55- bis 59-Jährigen aus 2030 angenommen und die Verschiebung für die 65- bis 69-Jährigen wird ebenso mit Bezug auf die 60- bis 64-Jährigen modelliert. Die Rentensimulation liefert sowohl für 2035 mit 2,4 Mio. zusätzlichen Erwerbspersonen als auch für 2060 mit noch 1,8 Mio. Personen quantitativ bedeutsame Mehrungen beim Erwerbspersonenpotenzial. Dagegen fallen die Zuwächse bei den Rechnungen zur Vereinbarkeit mit 0,6 Mio. 2035 und 0,5 Mio. 2060 sowie zur Integration mit 0,4 Mio. bzw. 0,3 Mio. deutlich schwächer aus. Der Gesamteffekt würde 2035 gut 3,4 Mio. und 2060 etwa 2,5 Mio. Erwerbspersonen betragen. Der Effekt schwächt sich wegen der demografischen Entwicklung ab, da die Erwerbsbeteiligung in Deutschland nach 2035 schon rein mathematisch nur noch wenig gesteigert werden kann. Inwieweit diese recht extremen Steigerungen der Erwerbsquoten wirklich möglich sind, wird hier nicht diskutiert.

Es zeigt sich, dass das Erwerbspersonenpotenzial in Deutschland voraussichtlich sinken wird. Selbst Szenarien mit sehr optimistischen Annahmen verdeutlichen, wie schwer es sein wird, diesen Trend zu verlangsamen. Allerdings bilden die Szenarien nur die Entwicklung auf der Personenebene ab. Volkswirtschaftlich sind auch die Arbeitszeiten wichtig, denn für das Wirtschaftswachstum ist das Arbeitsvolumen entscheidend. Längere Arbeitszeiten wären denkbar – immerhin arbeitet mehr als die Hälfte aller Arbeitnehmerinnen in Teilzeit (Wanger, 2020). Der daraus zu erwartende Effekt dürfte angesichts der Arbeitszeitwünsche der Beschäftigten jedoch gering sein: Im Durchschnitt möchten die Erwerbstätigen nur eine Stunde mehr pro Woche arbeiten. Ein stabiles Erwerbspersonenpotenzial erforderte jedoch eine Steigerung der Arbeitszeit um gut ein Drittel. Aufgrund der besonderen Bedeutung der Migration dürften politische Weichenstellungen wie das 2020 in Kraft getretene Fachkräfteeinwanderungsgesetz in die richtige Richtung zielen. Auch Bildungsmaßnahmen, steigende Qualifikation und damit eine höhere Produktivität der Beschäftigten können längerfristig die negativen Folgen eines sinkenden Erwerbspersonenpotenzials für Wirtschaft und Arbeitsmarkt mildern. Letztlich sind die aktive Teilnahme Älterer am Erwerbsleben, die Vereinbarkeit von Familie und Beruf sowie die Integration von Migrant:innen gesellschaftspolitische Ziele, die nicht nur aus Arbeitsmarktperspektive höchsten Stellenwert haben.

The demographic development in Germany will lead to a declining number of people of working age and thus potentially in the labour force. The age structure is shifting towards older cohorts. The labour force will decline by 16 million workers between 2020 and 2060. However, an increase in the labour force participation of women and elderly people combined with immigration will mitigate the demographic effect. An average net migration of 100,000 persons per year would result in a projected decline in the labour force of 9 million by 2060. An extremely high migration of at least 400,000 persons would allow the potential labour force to remain roughly constant. A sensitivity analysis also looks at the effects that may arise if the participation rates increased even further.

Dr. Johann Fuchs, Doris Söhnlein und Brigitte Weber sind Mitarbeitende des Forschungsbereichs „Prognosen und gesamtwirtschaftliche Analysen“ am IAB.


Literatur

Bauer, A., J. Fuchs, H. Gartner, M. Hummel, C. Hutter, S. Wanger, E. Weber und G. Zika (2021), IAB-Prognose: Arbeitsmarkt auf dem Weg aus der Krise, IAB-Kurzbericht, 6.
Fuchs, J., D. Söhnlein (2005): Vorausschätzung der Erwerbsbevölkerung bis 2050, IAB-Forschungsbericht, 16.
Fuchs J Weber B Höhere Erwerbsquoten stoppen nicht den Rückgang des Erwerbspersonenpotenzials Sozialer Fortschritt 2020 69 1 45 71 10.3790/sfo.69.1.45
Fuchs, J., D. Söhnlein und B. Weber (2021), Projektion des Erwerbspersonenpotenzials bis 2060: Demografische Entwicklung lässt das Arbeitskräfteangebot stark schrumpfen, IAB-Kurzbericht, 25.
Fuchs, J., B. Weber (2021): Neue Schätzungen für die Stille Reserve — erstmalig Anwendung des IAB-Konzepts auf Gesamtdeutschland. IAB-Forschungsbericht, 6.
Kubis, A. (2021), IAB-Stellenerhebung 3/2021: Zahl der offenen Stellen übertrifft mit 1,39 Millionen das Vorkrisenniveau, IAB-Forum, 9. November.
Wanger, S. (2020), Entwicklung von Erwerbstätigkeit, Arbeitszeit und Arbeitsvolumen nach Geschlecht, in Ergebnisse der IAB-Arbeitszeit-rechnung nach Alter und Geschlecht, IAB-Forschungsbericht, 16.
